# Tip-Sonication-Induced Dilute Alloying of Metal Pairs with Limited Bulk-Immiscibility

**DOI:** 10.3390/molecules31193371

**Published:** 2026-09-23

**Authors:** Hochan Ahn, Hojin Ahn

**Affiliations:** 1 Independent Researcher, Hwaseong-si 18472, Gyeonggi-do, Republic of Korea; dksghcks88@gmail.com; 2 Department of Chemistry, Konkuk University, Seoul 05029, Republic of Korea

**Keywords:** tip sonication, nonequilibrium alloying, immiscible metals, sonochemistry

## Abstract

Alloying metals with unfavorable bulk mixing thermodynamics remains challenging because conventional synthesis often favors elemental segregation rather than compositionally mixed nanoparticles. This study aimed to determine whether direct tip sonication can promote compositional intermixing in metal pairs with limited bulk miscibility and to distinguish sonication-specific effects from conventional thermal and chemical effects. Using Au–Mo as a model system, tip sonication of gold acetate and molybdenum hexacarbonyl in oleic acid successfully produced Au–Mo intermixed nanoparticles with an average diameter of 4.46 ± 0.89 nm and a bulk composition of 96.97 at% Au and 3.03 at% Mo. We also revealed that tip sonication alone was insufficient to induce alloy formation; rather, successful Au–Mo mixing required an appropriate precursor combination and solvent environment, highlighting the cooperative roles of physical energy input and reaction chemistry. The applicability of this strategy was further examined using other compositionally challenging metal pairs, including Ag–Rh and Au–Pt, for which tip sonication likewise produced bimetallic nanoparticles with spatially intermixed elemental distributions. Overall, these findings identify tip sonication as a simple nonequilibrium solution-phase route for accessing compositionally challenging dilute alloy nanoparticles.

## 1. Introduction

Alloy nanoparticles provide a versatile platform for tailoring the structural, electronic, optical, and catalytic properties of metals beyond those accessible from their monometallic counterparts [1,2]. Representative studies have demonstrated enhanced catalytic performance through surface and electronic structure control as well as composition-dependent optical properties [3,4]. At the nanoscale, composition, atomic ordering, surface segregation, and lattice strain can substantially alter electronic structures and surface chemical behavior [5,6,7]. Accordingly, extensive efforts have been devoted to synthesizing bimetallic and multimetallic nanoparticles with controlled compositions and elemental distributions [8,9,10]. However, the accessible compositional space remains constrained by bulk thermodynamics. For metal pairs with strongly unfavorable mixing, equilibrium synthesis tends to produce phase-separated structures rather than homogeneous alloys, limiting access to metastable atomic configurations that may exhibit distinct properties [11,12,13,14].

This challenge is particularly pronounced for metal combinations with unfavorable mixing thermodynamics and competing surface or strain effects, which can promote phase separation even at the nanoscale [12,13,14,15]. Au–Mo is a representative example, exhibiting only very limited mutual solubility in the equilibrium phase diagram and no broad stable substitutional solid solution [16]. Recent work has nevertheless shown that nanoscale dimensions can substantially alter miscibility behavior. For example, Chen et al. reported a size-dependent phase-separation-to-alloy transition in nominally immiscible Au–Rh nanoparticles, with complete miscibility emerging in the sub-2-nm regime [17]. These observations highlight that nanoscale mixing can deviate markedly from bulk equilibrium behavior, but accessing such mixed states generally requires kinetic or size-dependent stabilization.

A variety of nonequilibrium strategies have been developed to address this problem. Rapid co-reduction has enabled solid-solution nanoparticles from otherwise immiscible or poorly miscible systems such as Ag–Rh [18], Pd–Ru [19], and Ru–Cu [20]. These approaches benefit from solution-phase processability, but homogeneous alloy formation often depends sensitively on synchronizing the reduction, nucleation, and growth kinetics of chemically dissimilar precursors. Differences in redox potentials, ligand coordination, precursor decomposition, or nucleation rates can readily lead to sequential reduction, surface segregation, core–shell growth, or separate phases. Recent mechanistic studies of colloidal multimetallic nanoparticles have likewise emphasized that precursor reaction pathways and reduction kinetics strongly influence the final compositional homogeneity [21]. Other nonequilibrium methods can also access compositions that are difficult to obtain by conventional wet chemistry. These include sputtering-based approaches [22], laser-induced processing [23], and microwave-assisted synthesis of classically immiscible metal combinations [24,25]. Such methods, however, often require specialized instrumentation or tightly controlled transient processing conditions. A notable example is the carbothermal shock method reported by Yao et al., in which rapid high-temperature treatment followed by ultrafast cooling kinetically trapped multielement alloy nanoparticles that are difficult to access under equilibrium conditions [9]. More broadly, recent reviews of nonequilibrium nanomaterial synthesis have emphasized that rapid kinetic trapping can substantially expand the compositional space available beyond equilibrium miscibility constraints [26].

High-intensity ultrasound offers a conceptually distinct route to nonequilibrium materials synthesis. Its effects in liquids originate primarily from acoustic cavitation, in which bubbles nucleate, grow, and collapse, concentrating acoustic energy into short-lived local environments with extreme temperature and pressure gradients while the bulk liquid remains at a much lower temperature [27,28,29]. These physical effects can promote particle-surface restructuring and enhanced dispersion or mass transport [30,31]. Ultrasound can also induce nanoparticle collision and fusion and generate strong interfacial effects at solid–liquid boundaries [32,33]. Sonochemical routes have consequently been applied to the synthesis of metallic nanoparticles [34,35] and engineered bimetallic nanostructures [36,37]. Hayashi et al. demonstrated rapid Au–Ag solid-solution formation within 10 min of ultrasonic irradiation, directly illustrating the potential of ultrasound to promote alloying [38]. Ultrasound-assisted synthesis has also been extended to other bimetallic systems, including Pd–Co nanoparticles [39], while recent reviews have summarized the growing use of acoustic cavitation in colloidal nanocrystal synthesis [40,41,42].

Despite these advances, the deliberate use of direct tip sonication to promote dilute compositional intermixing in metal pairs with strongly limited bulk miscibility remains comparatively underexplored. The key question of this study is whether direct tip sonication can promote intermixing in a strongly limited-miscibility system such as Au–Mo, and whether this effect can be distinguished from conventional thermal reduction caused by the macroscopic temperature rise during sonication. In addition, the extent to which precursor identity and solvent environment cooperate with the nonequilibrium physical effects of tip sonication remains insufficiently clarified. Accordingly, the objective of this study was to determine whether direct tip sonication can promote compositional intermixing in metal pairs with limited bulk miscibility and to disentangle the roles of the nonequilibrium sonication environment, bulk thermal effects, precursor identity, and solvent chemistry. Using Au–Mo as a model system, we demonstrate that tip sonication enables the formation of Au–Mo nanoparticles in which Mo is incorporated at dilute concentrations of approximately 3 at%. Importantly, reproducing the macroscopic temperature reached during sonication by conventional heating did not result in comparable Au–Mo mixing, indicating that the observed Au–Mo intermixing cannot be attributed solely to bulk thermal effects. We further show that altering either the precursor combination or the solvent suppresses effective Au–Mo mixing even under otherwise identical sonication conditions. These results suggest that tip sonication provides a nonequilibrium physical environment that facilitates intermixing, while appropriate precursor and solvent chemistries establish conditions favorable for the formation and stabilization of the compositionally intermixed Au–Mo state. The transferability of this approach was further explored using Ag–Rh and Au–Pt as additional compositionally challenging systems, which exhibited different degrees of sonication-induced compositional intermixing. Collectively, these findings suggest that tip sonication can provide a simple solution-phase route for promoting compositional intermixing beyond conventional bulk miscibility constraints and provide a basis for expanding nonequilibrium alloy synthesis to a broader range of challenging metal combinations.

## 2. Results and Discussion

### 2.1. Formation of Dilute Au–Mo Alloy Nanoparticles

Figure 1a illustrates the synthetic concept for the tip-sonication-induced compositional intermixing in Au–Mo nanoparticles. Gold acetate and molybdenum hexacarbonyl were used as the Au and Mo precursors, respectively, and were subjected directly to high-intensity tip sonication in oleic acid. Rather than employing ultrasound merely as a mixing step, nanoparticle formation was carried out under direct sonication so that the reaction proceeded under cavitation-driven nonequilibrium conditions. Under the optimized precursor and solvent conditions, this process produced dispersible Au-rich nanoparticles containing a small fraction of Mo.

The representative transmission electron microscopy (TEM) image in Figure 1b shows that the resulting Au–Mo nanoparticles (AuMo NPs) exhibited a nearly spherical morphology. Particle-size analysis of 152 nanoparticles collected from three independently prepared synthesis batches gave an average diameter of 4.46 ± 0.89 nm, with the corresponding size-distribution histogram provided in Figure S1. The high-angle annular dark-field-scanning TEM (HAADF-STEM) image (Figure 1c) and energy-dispersive X-ray spectroscopy (EDS) elemental mapping images (Figure 1d–f) showed spatial overlap of Au and Mo signals within individual particles. No obvious Mo-rich domains were observed within the spatial resolution of the EDS mapping. To further evaluate the local compositional reproducibility, quantitative EDS analyses were performed at three independently selected nanoparticle-containing regions. The average Au and Mo contents determined by EDS were 95.36 ± 0.23 at% and 4.64 ± 0.23 at%, respectively, with only a small variation among the three measurements. A representative HAADF-STEM image and corresponding EDS spectrum are provided in Figure S2. Bulk elemental quantification by ICP-OES gave a composition of 96.97 at% Au and 3.03 at% Mo. The resulting composition therefore lies in a dilute Au-rich regime rather than representing an equiatomic or compositionally broad solid solution. To determine the fate of the initially added metals, the precipitated fraction and supernatant obtained after the first centrifugation were separately analyzed by ICP-OES. Relative to the initially introduced metal amounts, 91.4% of Au and 2.81% of Mo were recovered in the precipitated fraction, whereas 6.5% of Au and 94.2% of Mo remained in the supernatant. These results indicate that Au is preferentially incorporated into the isolated nanoparticle fraction, whereas most of the Mo remains in the solution phase, accounting for the dilute Mo content of the final Au-rich nanoparticles.

X-ray photoelectron spectroscopy (XPS) was employed to examine the chemical states and electronic interactions of Au and Mo in the AuMo NPs (Figure 2). The Au 4f spectrum exhibited the characteristic doublet of metallic Au, confirming that Au predominantly remained in the metallic state. More importantly, deconvolution of the Mo 3d spectrum revealed contributions from both oxidized and metallic Mo species. The metallic Mo component accounted for approximately 30.3% of the Mo 3d signal, while the remaining contribution was assigned to oxidized Mo species. The oxidized component is tentatively attributed to oxidation of surface-exposed Mo species upon exposure to air, whereas the metallic component indicates that a substantial fraction of detected Mo remains in a reduced state within the nanoparticles. Metallic Mo states have also been identified by XPS in previously reported PdMo bimetallene systems [43,44], demonstrating that reduced Mo can be stabilized in close association with a noble-metal matrix. In the present system, the metallic Mo component therefore supports the presence of reduced Mo species in close association with Au when considered together with the EDS results. Notably, clear binding energy shifts were observed for both Au and Mo relative to their corresponding reference states. The Au 4f_7/2_ peak shifted from 84.0 to 84.5 eV, whereas the Mo^6+^ 3d_5/2_ and metallic Mo components shifted from 233.1 to 232.7 eV and from 228.0 to 226.4 eV, respectively. Although the relatively large binding-energy shift in Mo should be interpreted cautiously, the opposite shifts in the Au and Mo core levels suggest modification of their local electronic environments associated with Au–Mo intermixing, consistent with electronic interactions between Au and Mo. These changes further support intimate interaction between the two constituent metals rather than the simple coexistence of electronically independent Au and Mo species.

Powder X-ray diffraction (XRD) patterns were obtained to identify the crystal structure of the AuMo NPs. The diffraction pattern was dominated by reflections characteristic of the face-centered cubic (fcc) Au lattice, with no additional crystalline phases detected (Figure 3a). Given the low Mo fraction, the absence of distinct Mo-derived diffraction peaks does not by itself exclude the presence of a minor segregated or poorly crystalline Mo-containing phase, but it is consistent with the absence of a substantial crystalline Mo-rich fraction. Compared with the bulk fcc Au, the diffraction peaks of the AuMo NPs were slightly shifted toward lower 2*θ* values, indicating an increase in the average lattice spacing (Figure 3b). This lattice expansion may reflect local lattice distortion and strain associated with the nonequilibrium incorporation of Mo into the Au lattice. Taken together, the EDS, XPS, ICP-OES, and XRD results support nanoscale compositional intermixing of Au and Mo in Au-rich nanoparticles that retain a Au-dominant fcc crystal structure.

### 2.2. Roles of Sonication and Reaction Chemistry in Au–Mo Alloy Formation

To distinguish the specific effects of tip sonication from those of conventional thermal heating, a control experiment was conducted. During the standard tip sonication procedure, the temperature of the bulk solution was measured to rise to approximately 105 °C. To isolate the contribution of this macroscopic temperature rise, the AuMo precursor mixture was heated conventionally to 105 °C using an oil bath without tip sonication. The TEM image of the resulting nanoparticles showed a morphology similar to that of the tip-sonicated AuMo NPs (Figure 4a). EDS analysis detected Au but no measurable Mo signal; the corresponding Au map is shown in Figure 4b. The Au 4f spectrum of the thermally treated sample exhibited the characteristic Au 4f_7/2_ and Au 4f_5/2_ peaks at 84.0 and 87.7 eV, respectively, confirming the formation of metallic Au under conventional heating conditions (Figure 4c). In contrast to the tip-sonicated AuMo NPs, however, the Au 4f_7/2_ peak did not exhibit the positive shift observed for the AuMo NPs. More importantly, no discernible Mo 3d signal was detected over the corresponding binding energy range (Figure 4d), indicating that conventional heating under these conditions did not result in detectable incorporation of Mo into the resulting nanoparticles. A quantitative ICP-OES comparison further supported this difference. Under conventional heating, 90.8% of the initially added Au but only 0.2% of Mo were recovered in the precipitated fraction, while 7.8% of Au and 97.9% of Mo remained in the supernatant. Thus, recovery was comparable between tip sonication and conventional heating, whereas Mo recovery in the precipitated fraction was markedly higher under tip sonication. The trace amount of Mo detected in the precipitate may partly originate from residual mother liquor retained after the first centrifugation step. Taken together, these results show that the macroscopic temperature increase accompanying tip sonication is sufficient to promote Au reduction but is not sufficient to reproduce the Au–Mo mixed state. The distinct Au core-level shift and detectable metallic Mo observed only after tip sonication therefore support a specific role of the sonication environment in enabling Mo incorporation and electronic interaction between Au and Mo.

To further determine whether Au–Mo mixing was governed solely by the physical environment generated by tip sonication, additional control experiments were performed by varying the precursor combination and solvent. First, each metal precursor was independently subjected to the same tip sonication conditions used for the AuMo NPs synthesis. When only the Au precursor was present, nanoparticles were still formed, but they exhibited larger sizes and highly irregular, aggregated morphologies compared with the relatively uniform AuMo NPs (Figure 5a). EDS analysis detected only Au, as expected in the absence of the Mo precursor (Figure 5b). In contrast, sonication of the Mo precursor alone did not yield any isolable solid product after centrifugation. These observations indicate that the Au precursor can independently undergo nanoparticle formation under tip sonication, whereas the Mo precursor does not readily form Mo-containing nanoparticles under the same conditions. The markedly different morphology obtained in the absence of the Mo precursor further suggests that the presence of both precursors influences the nucleation and growth behavior of the nanoparticles.

When gold acetate was replaced with gold(I) chloride, substantially larger nanoparticles were obtained (Figure 6a). HAADF-STEM and EDS analyses further revealed that Au and Mo were not homogeneously intermixed within the particles but instead formed spatially separated regions (Figure 6b,c). Consistent with this observation, the Au 4f_7/2_ peak appeared at 84 eV without the positive binding energy shift observed for the AuMo NPs synthesized from gold acetate (Figure 6d). Moreover, the Mo 3d spectrum showed only the Mo^6+^ component, while the metallic Mo component characteristic of the optimized AuMo NPs was absent (Figure 6e). These structural and spectroscopic differences demonstrate that replacing the Au precursor substantially alters the nanoparticle formation pathway and suppresses effective incorporation of metallic Mo into the particles. The results therefore indicate that precursor identity is an important chemical parameter for achieving the dilute Au–Mo mixed state under tip sonication, rather than tip sonication alone being sufficient to produce the compositionally intermixed Au–Mo state.

A similarly strong dependence was observed for the solvent environment. When oleic acid was replaced with octadecene while the precursor combination and tip sonication conditions were otherwise maintained, nanoparticles with an overall morphology similar to that of the optimized AuMo NPs were obtained (Figure 7a). Despite their morphological similarity, however, their surface electronic structure differed markedly from that of the AuMo NPs prepared in oleic acid. The Au 4f_7/2_ peak remained near 84 eV and showed no measurable positive shift (Figure 7b), while the Mo 3d spectrum exhibited only Mo^6+^, with no detectable metallic Mo component (Figure 7c). These results suggest that oleic acid plays a role beyond serving as a liquid medium, providing a chemical environment that favors the incorporation and stabilization of reduced Mo species within the Au-rich nanoparticles.

Taken together with the thermal heating control, these experiments show that dilute Au–Mo alloy formation cannot be explained simply by the macroscopic temperature increase or physical energy input associated with tip sonication. Tip sonication provides the nonequilibrium environment necessary for the reaction, but successful Au–Mo mixing additionally requires an appropriate combination of metal precursors and solvent chemistry. The strong sensitivity to both precursor and solvent therefore points to a coupled physical–chemical process in which cavitation-driven energy input and reaction kinetics act together to enable the formation of the dilute nonequilibrium Au–Mo mixed state. Au–Mo mixing has previously been achieved under other nonequilibrium conditions. Sun et al. synthesized Mo-doped Au nanoparticles by a solvothermal method at 280 °C, obtaining nanoparticles with a Mo/Au atomic ratio of 8.7% [45]. Earlier studies also demonstrated Mo–Au surface alloying by physical vapor deposition or molecular beam epitaxy at elevated temperatures [46,47]. In comparison, the present approach produces Au-rich nanoparticles containing 3.03 at% Mo through direct tip sonication, with the bulk solution temperature reaching approximately 105 °C. These results demonstrate an alternative solution-phase route for accessing dilute Au–Mo intermixing under substantially milder bulk thermal conditions.

### 2.3. Extension of Tip-Sonication-Induced Mixing to Other Metal Pairs

To examine whether the tip sonication approach could be extended beyond the Au–Mo model system, additional metal combinations with unfavorable equilibrium mixing tendencies were investigated. Ag–Rh represents a strongly phase-separating system despite both metals adopting fcc crystal structures, reflecting unfavorable heteroatomic interactions and mixing thermodynamics [18,48]. For Au–Pt, both metals also possess fcc lattices but exhibit a miscibility gap and a pronounced tendency toward elemental segregation under equilibrium conditions [11,49].

The HAADF-STEM image of the resulting Ag–Rh nanoparticles is shown in Figure 8a, together with the corresponding EDS elemental maps in Figure 8b–d. The Ag and Rh signals were spatially distributed throughout the same nanoparticles, with no obvious large Ag-rich or Rh-rich regions observed. The overlap of the two elemental distributions is consistent with nanoscale mixing of Ag and Rh rather than the formation of clearly separated monometallic particles or large phase-segregated domains. Quantitative EDS analysis gave an average composition of 96.88 at% Ag and 3.12 at% Rh. In addition, a lower-magnification HAADF-STEM image and corresponding EDS elemental mapping image showed consistent co-localization of Ag and Rh across a broader nanoparticle population (Figure S3a,b). The Au–Pt system exhibited a more heterogeneous morphology and elemental distribution. The HAADF-STEM image in Figure 8e and corresponding EDS elemental maps (Figure 8f–h) showed spatial overlap of Au and Pt within the analyzed nanoparticle, indicating local compositional intermixing. Quantitative EDS analysis yielded an average composition of 89.9 at% Au and 10.1 at% Pt. However, a lower-magnification HAADF-STEM image and corresponding EDS elemental mapping image revealed a heterogeneous nanoparticle population, in which Au and Pt were co-localized in many regions while Au- and Pt-enriched particles or regions were also observed (Figure S3c,d). Thus, under the present conditions, tip sonication promotes partial Au–Pt intermixing but does not produce a uniformly intermixed nanoparticle population. Although EDS mapping and quantitative EDS analysis do not establish atomic-scale alloying, the Ag–Rh results show consistent nanoscale co-localization of the constituent metals, whereas the Au–Pt results demonstrate partial and heterogeneous compositional intermixing. These findings provide proof-of-concept evidence that tip sonication can facilitate compositional intermixing in additional metal systems.

## 3. Materials and Methods

### 3.1. Chemicals and Materials

Molybdenum hexacarbonyl (Mo(CO)_6_, 99.9% trace metal basis), Gold(I) chloride (AuCl, 99.9% trace metal basis), Oleic acid (90%), 1-Octadecene (90%), Ethanol (99.5%), Hexane (95%, anhydrous), Silver(I) nitrate (AgNO_3_, 99.0%), Rhodium(III) acetylacetonate (Rh(acac)_3_, 97%), Ethylene glycol (EG, 99.8%, anhydrous), Gold(III) chloride trihydrate (HAuCl_4_·3H_2_O, 99.9% trace metals basis), Poly(vinylpyrrolidone) (PVP, average M_w_ = 55,000), Platinum(II) acetylacetonate (Pt(acac)_2_, 97%), 1,5-pentanediol (96%) were purchased from Sigma-Aldrich (St. Louis, MO, USA). Gold(III) acetate (99.9% metal basis) was obtained from Alfa Aesar (Ward Hill, MA, USA). All chemicals were used as received without further purification.

### 3.2. Synthesis of the AuMo NPs

In a typical synthesis of the AuMo NPs, 26.4 mg of Mo(CO)_6_ and 20 mL of oleic acid were mixed and stirred for 24 h to yield a homogeneous solution of Mo(CO)_6_ with a concentration of 5 mM. Subsequently, gold(III) acetate was dissolved in 10 mL of the Mo(CO)_6_ solution to achieve a homogeneous precursor solution with final concentrations of 5 mM for both Au and Mo. The resulting mixture was subjected to ultrasonic irradiation using an ultrasonic processor (Branson SFX550 Sonifier (Brookfield, CT, USA) equipped with a 1/8-inch tapered microtip) at 30% amplitude with a pulse mode of 1 s on and 1 s off. The ultrasonic irradiation continued until the total irradiation time reached 10 min. The colloidal product was collected by centrifugation and washed two times with a mixture of ethanol and hexane to remove the excess oleic acid. The final product was dispersed in hexane for further use.

The gold acetate/oleic acid combination was selected based on the control experiments described above, which showed that changes in either the Au precursor or solvent suppressed effective Au–Mo intermixing. A pulsed sonication program was used to provide effective cavitation while limiting excessive bulk heating.

### 3.3. Synthesis of the AgRh NPs

A PVP solution (5 mg mL^−1^) was first prepared in EG. AgNO_3_ and Rh(acac)_3_ precursor solutions were then prepared separately in the PVP-containing EG solution, each at a concentration of 1 mM. Subsequently, 5 mL of the AgNO_3_ solution and 5 mL of the Rh(acac)_3_ solution were mixed to obtain a homogeneous precursor solution. The resulting mixture was subjected to ultrasonic irradiation using the ultrasonic processor operated at 30% amplitude in a pulsed mode of 1 s on and 1 s off. The ultrasonic irradiation continued until the total irradiation time reached 10 min. The product was collected by centrifugation, washed two times with ethanol, and dispersed in ethanol for future use.

PVP-containing ethylene glycol was used to provide colloidal stabilization during nanoparticle formation, while the same sonication conditions used for the AuMo synthesis were maintained to evaluate the extension of the approach under a consistent ultrasonic input.

### 3.4. Synthesis of the AuPt NPs

A PVP solution (5 mg mL^−1^) was first prepared in 1,5-pentanediol. HAuCl_4_·3H_2_O and Pt(acac)_2_ precursor solutions were then prepared separately in the PVP-containing 1,5-pentanediol solution, each at a concentration of 1 mM. Subsequently, 5 mL of the HAuCl_4_·3H_2_O solution and 5 mL of the Pt(acac)_2_ solution were combined and mixed thoroughly to obtain a homogeneous precursor solution. The resulting mixture was subjected to ultrasonic irradiation using the ultrasonic processor operated at 30% amplitude in a pulsed mode of 1 s on and 1 s off. Ultrasonic irradiation continued until the total irradiation time reached 10 min. The product was collected by centrifugation, washed two times with ethanol, and dispersed in ethanol for future use.

### 3.5. Characterization

TEM images were obtained with an FEI Tecnai F30 Super-Twin transmission electron microscope (Hillsboro, OR, USA) operated at an accelerating voltage of 300 kV. HAADF–STEM imaging and EDS elemental mapping were performed on an FEI Talos F200X microscope at 200 kV or on a JEM-ARM200F (Tokyo, Japan) at 200 kV. For TEM analysis, the nanoparticle dispersions were drop-cast onto carbon-coated copper grids (300 mesh) and allowed to dry prior to measurement. XPS spectra were collected by a Thermo VG Scientific Sigma Probe spectrometer (Thermo Fisher Scientific, Waltham, MA, USA) equipped with an Al Kα X-ray source (1486.7 eV), and the binding energies were referenced to the C 1s signal at 284.8 eV. XRD measurements were conducted using a RIGAKU Ultima IV thin-film X-ray diffractometer (Tokyo, Japan). The elemental compositions of the samples were quantified by ICP-OES using an Agilent 5110 ICP OES (Santa Clara, CA, USA).

## 4. Conclusions

Tip sonication produced Au-rich nanoparticles with an average size of 4.46 ± 0.89 nm and a bulk composition of 96.97 at% Au and 3.03 at% Mo. Under tip sonication, 2.81% of the initially added Mo was recovered in the nanoparticle fraction, compared with only 0.2% under conventional heating at the same maximum bulk temperature of 105 °C. Control experiments further showed that effective Au–Mo intermixing depended strongly on both precursor identity and solvent environment, indicating a coupled contribution of the nonequilibrium sonication environment and reaction chemistry. Extension to Ag–Rh and Au–Pt provided additional proof-of-concept evidence for tip-sonication-induced compositional intermixing, although the degree of mixing was system-dependent. Overall, this study presents a simple solution-phase strategy for accessing dilute alloys beyond conventional bulk miscibility constraints, thereby offering the potential for expansion to a broader range of alloy systems in the future.

## Figures and Tables

**Figure 1:**
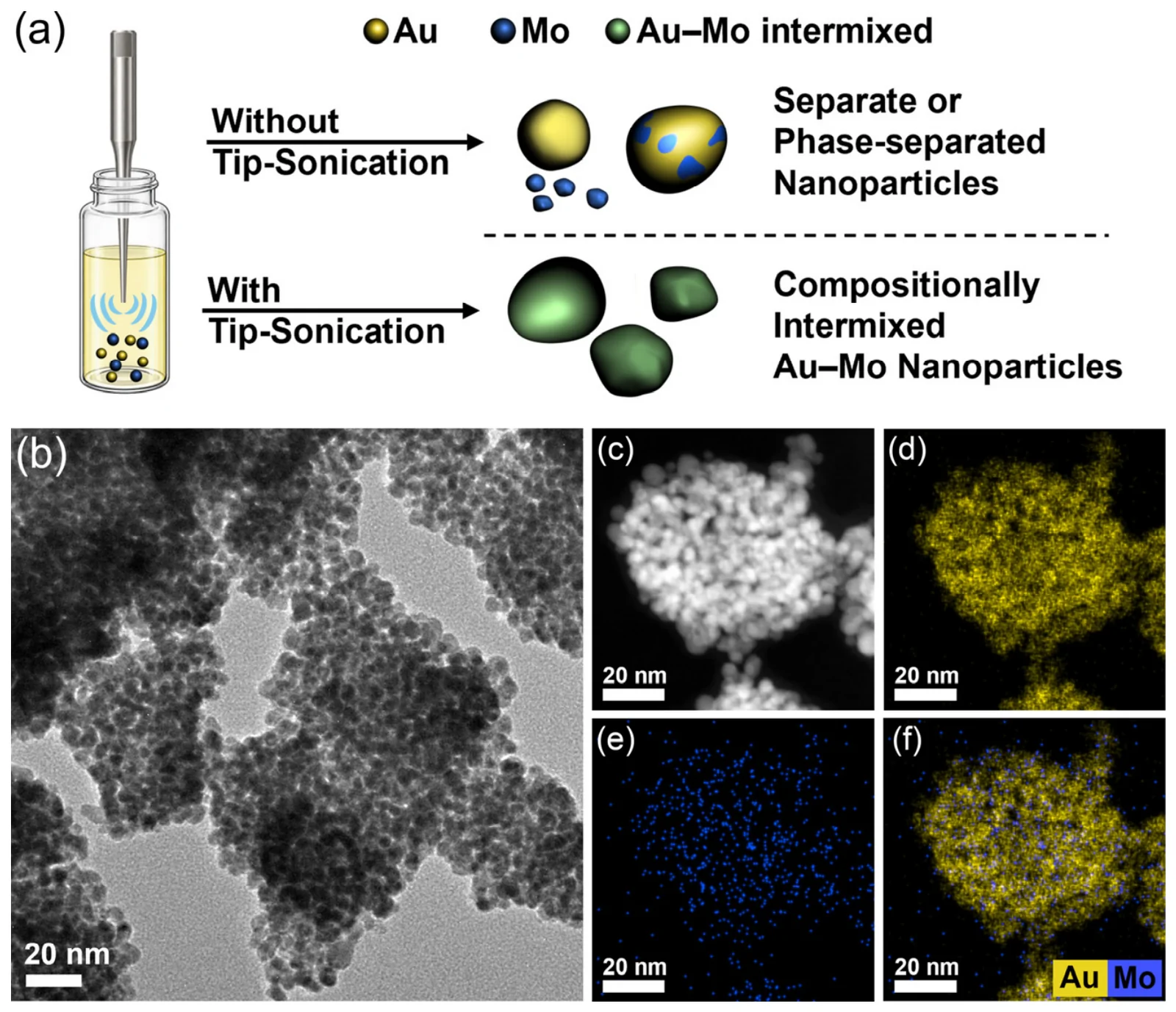
(**a**) Schematic illustration of tip-sonication-induced formation of compositionally intermixed Au–Mo nanoparticles. (**b**) TEM image of the AuMo NPs. (**c**) HAADF-STEM image and corresponding EDS elemental maps of (**d**) Au and (**e**) Mo, and (**f**) the overlaid Au–Mo distribution.

**Figure 2:**
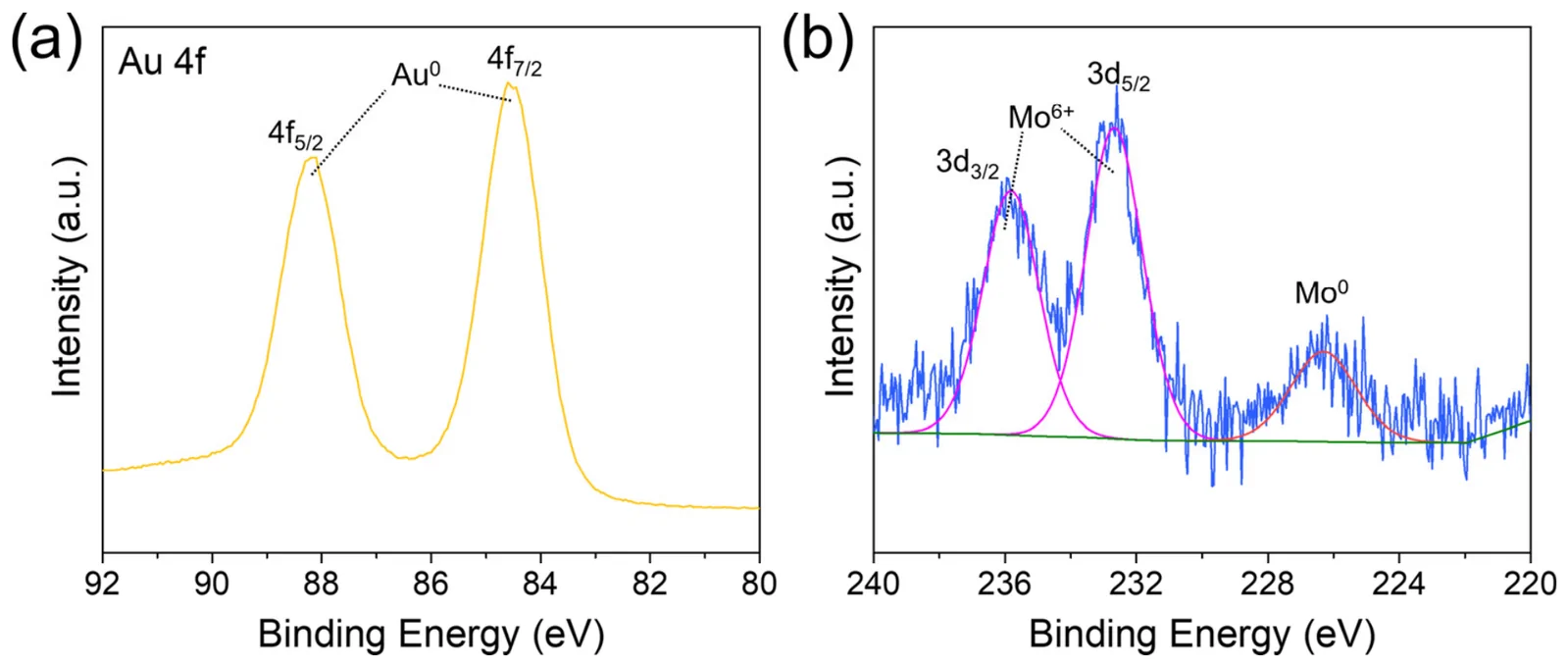
(**a**) Au 4f and (**b**) Mo 3d XPS spectra of the AuMo NPs.

**Figure 3:**
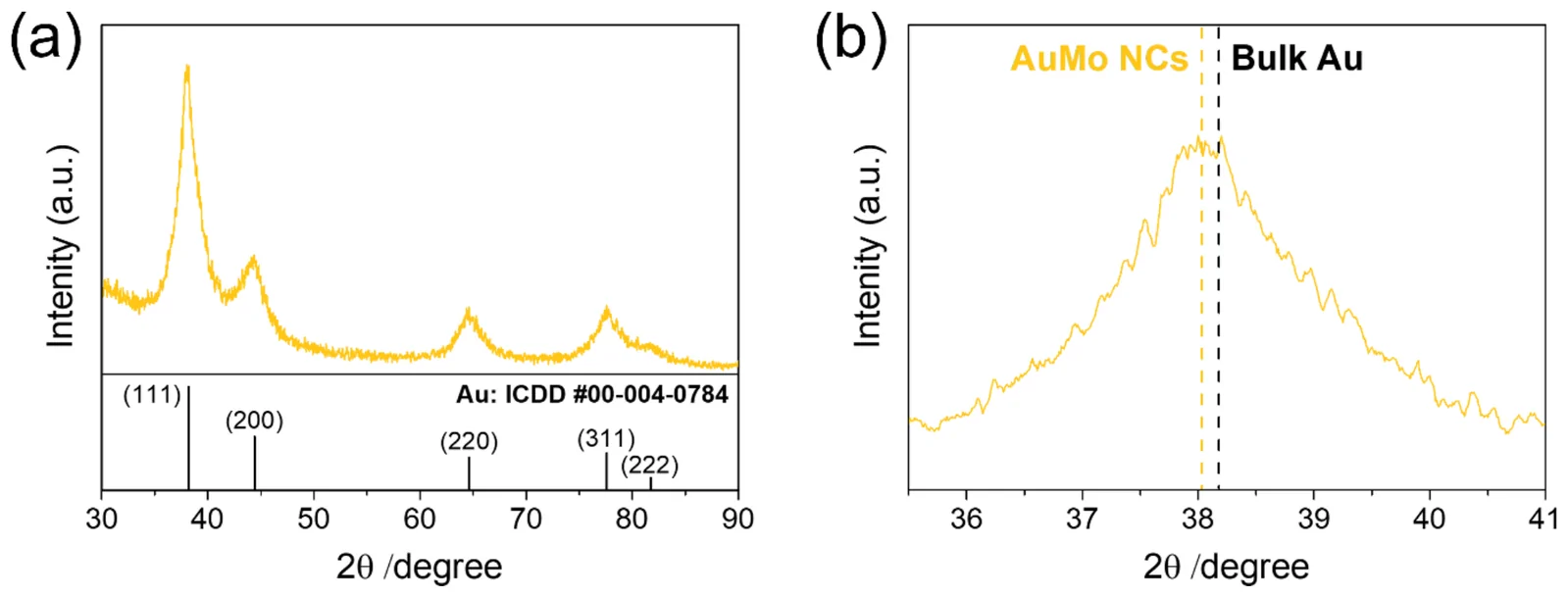
(**a**) XRD pattern of the AuMo NPs with the reference diffraction positions of fcc Au (ICDD No. 00-004-0784). (**b**) Enlarged Au(111) diffraction region comparing the AuMo NPs with bulk Au.

**Figure 4:**
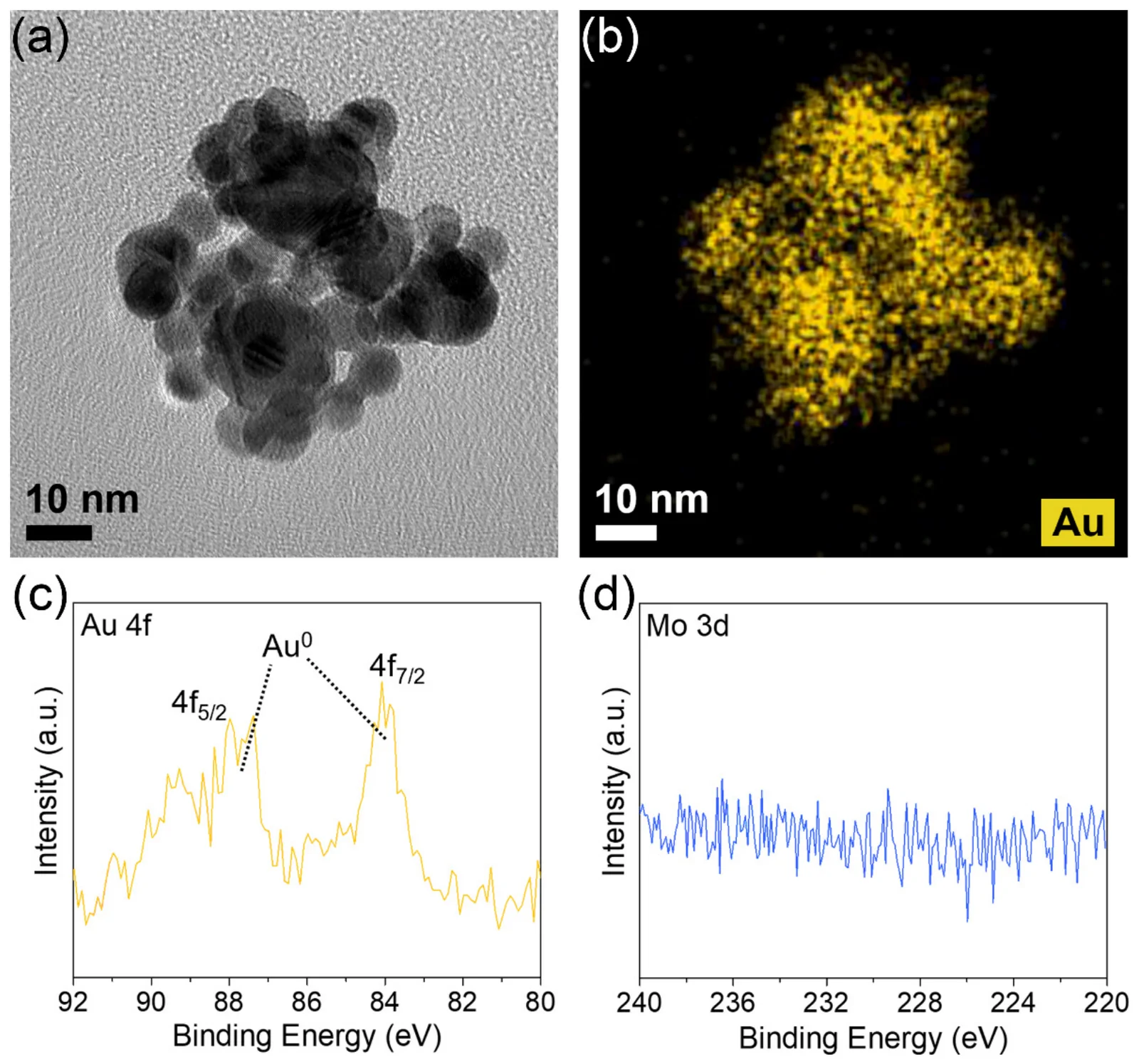
The standard AuMo precursor mixture was heated to 105 °C in an oil bath, corresponding to the maximum bulk temperature measured during tip sonication, without ultrasonic irradiation; all other precursor and solvent conditions were maintained as in the standard AuMo synthesis. (**a**) TEM image and (**b**) corresponding Au EDS elemental map of the resulting particles. XPS spectra of the (**c**) Au 4f and (**d**) Mo 3d regions.

**Figure 5:**
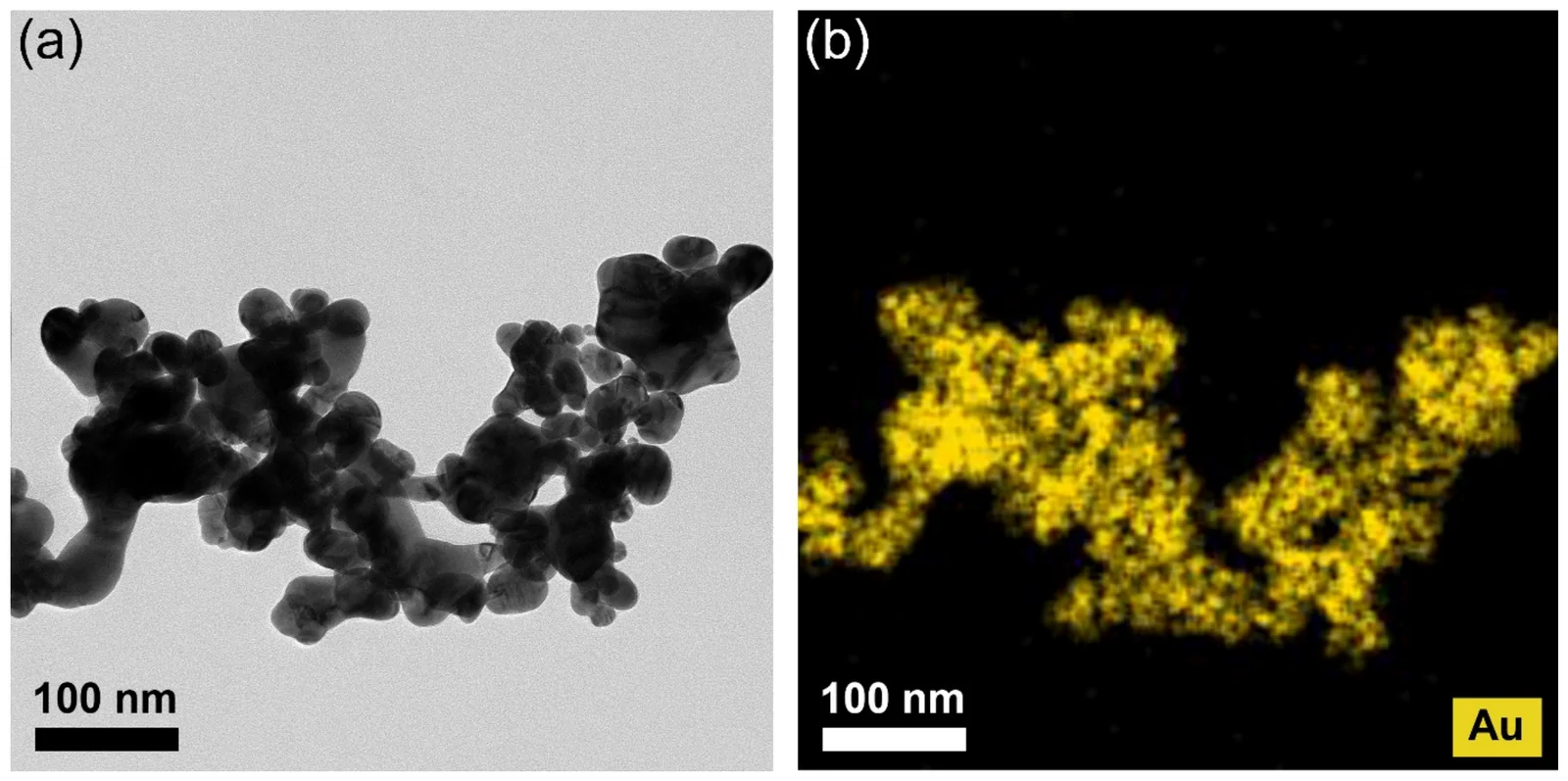
The reaction was performed under the standard AuMo NPs synthesis conditions except that Mo(CO)_6_ was excluded from the precursor solution. (**a**) TEM image and (**b**) corresponding EDS elemental map of the resulting Au nanoparticles.

**Figure 6:**
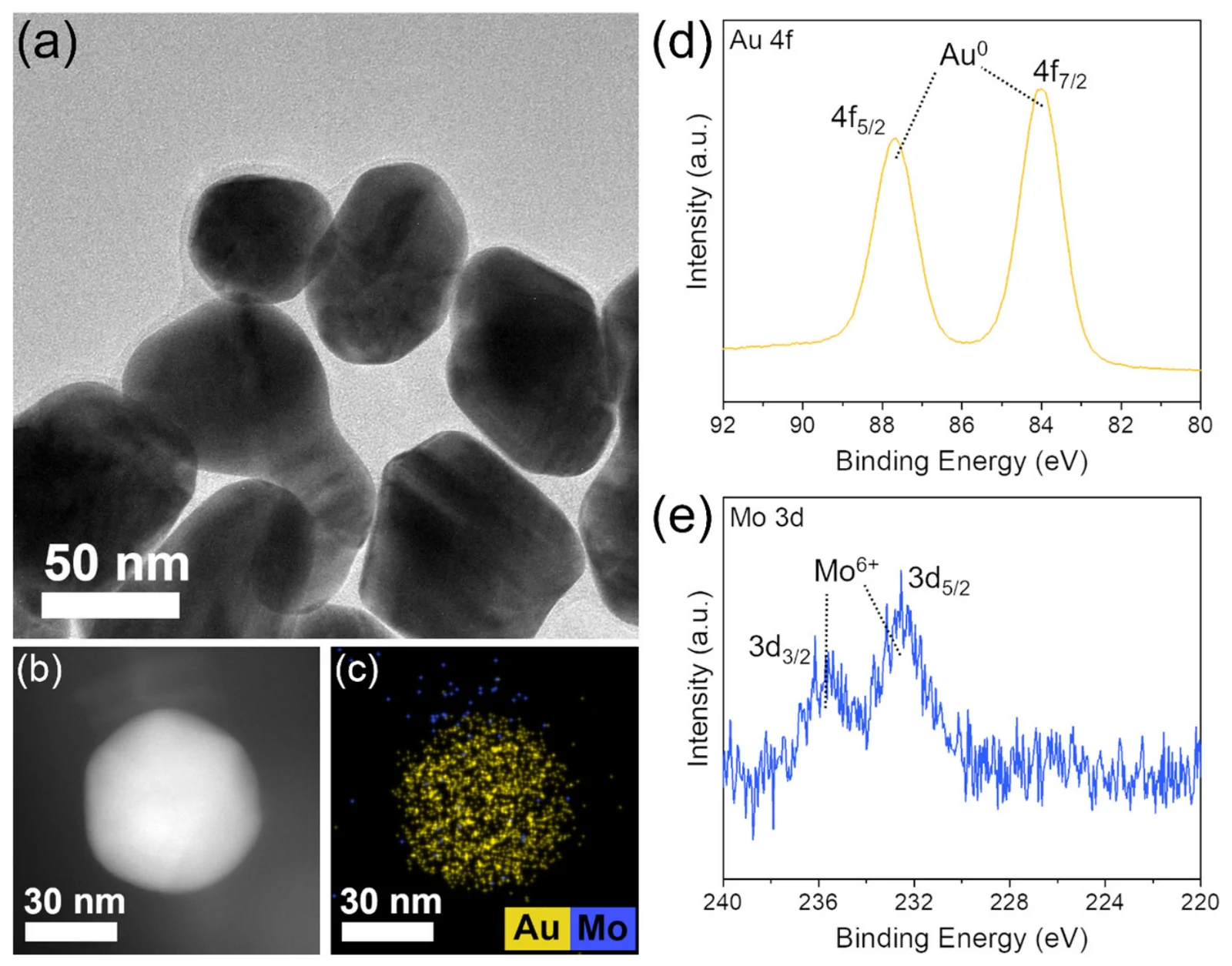
The reaction was performed under the standard AuMo synthesis conditions except that gold(III) acetate was replaced with AuCl, while the other reaction conditions were kept identical. (**a**) TEM image, (**b**) HAADF-STEM image, and (**c**) corresponding overlaid Au–Mo EDS elemental map of the resulting particles. XPS spectra of the (**d**) Au 4f and (**e**) Mo 3d regions.

**Figure 7:**
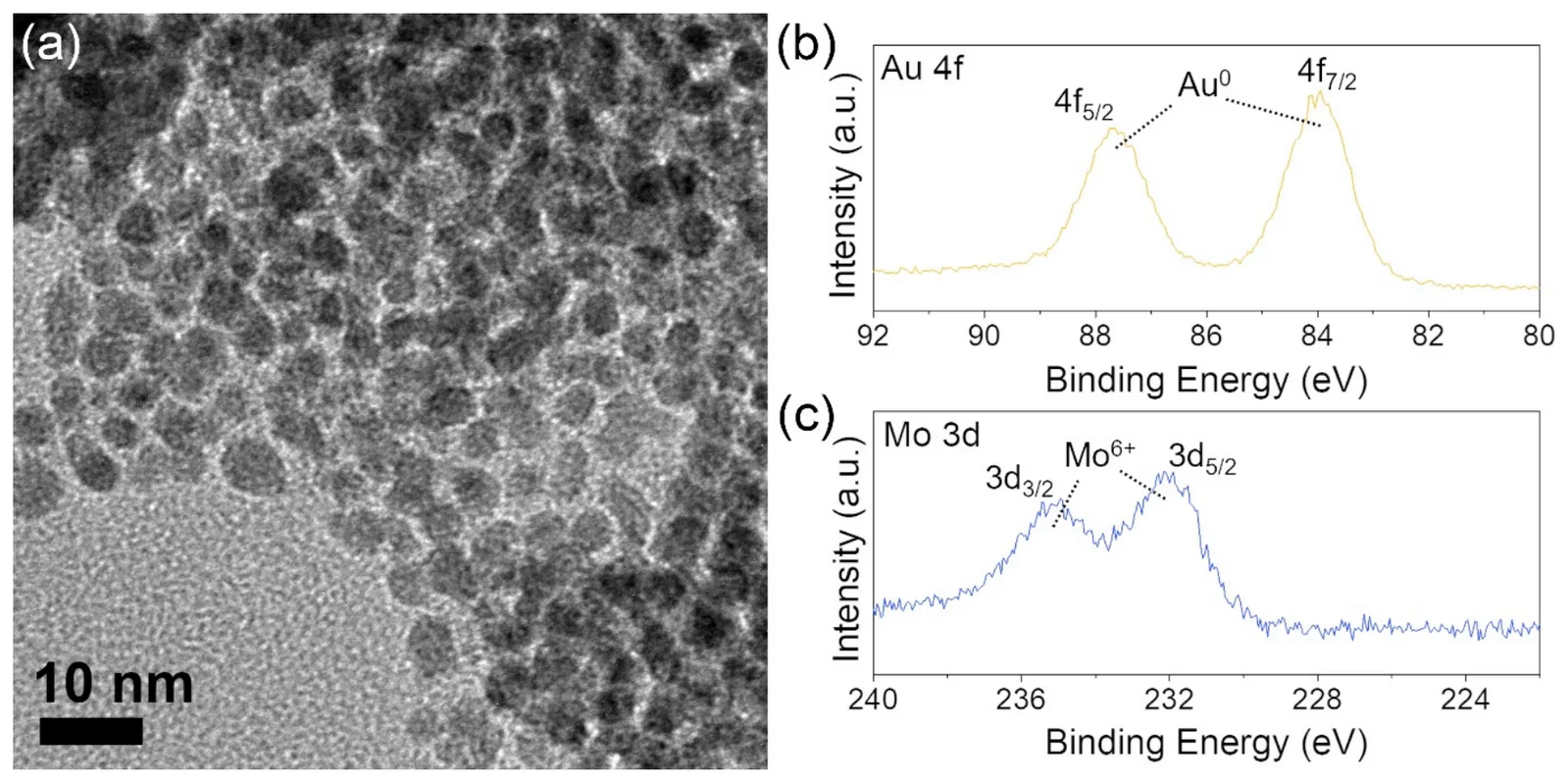
The reaction was performed under the standard AuMo NPs synthesis conditions except that oleic acid was replaced with 1-octadecene while the other conditions were kept identical. (**a**) TEM image of the resulting nanoparticles and XPS spectra of the (**b**) Au 4f and (**c**) Mo 3d regions.

**Figure 8:**
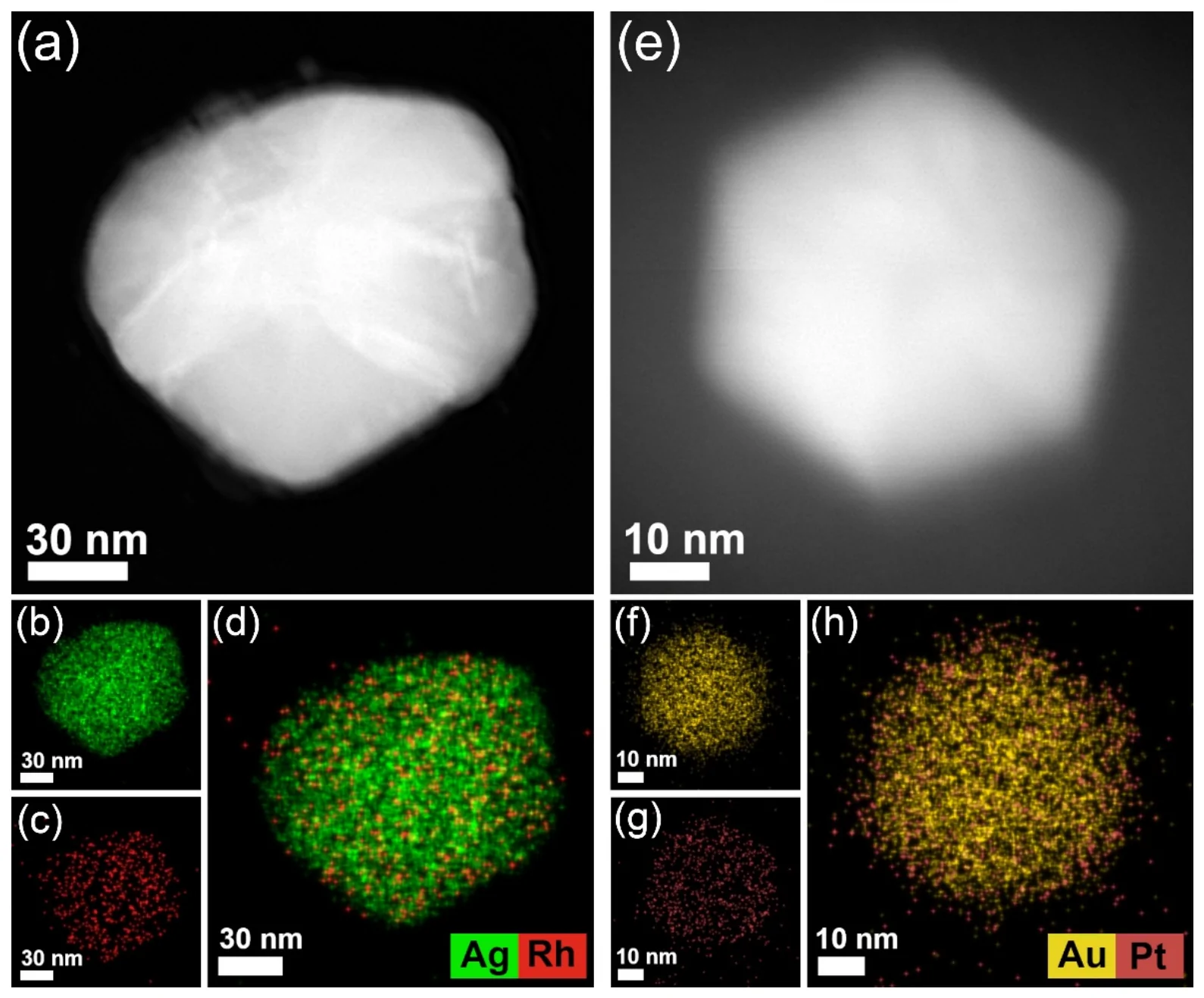
(**a**) HAADF-STEM image of an Ag–Rh nanoparticle and corresponding elemental maps of (**b**) Ag and (**c**) Rh, and (**d**) the overlaid Ag–Rh distribution. (**e**) HAADF-STEM image of a Au–Pt nanoparticle and corresponding elemental maps of (**f**) Au and (**g**) Pt, and (**h**) the overlaid Au–Pt distribution.

## Data Availability

The original contributions presented in this study are included in the article and Supplementary Materials. Further inquiries can be directed to the corresponding author.

## Supplementary Materials

The following supporting information can be downloaded at https://www.mdpi.com/article/10.3390/molecules31193371/s1. Figure S1 shows the particle-size distribution of the AuMo NPs; Figure S2 shows a representative HAADF-STEM image and corresponding EDS spectrum of the AuMo NPs; Figure S3 shows lower-magnification HAADF-STEM images and corresponding EDS elemental maps of the Ag–Rh and Au–Pt nanoparticles.
