# Supplementary material for: Tip-Sonication-Induced Dilute Alloying of Metal Pairs with Limited Bulk-Immiscibility
Source: Molecules. 2026 Sep 23;31(19):3371. doi: 10.3390/molecules31193371 (PMC13666483; doi:10.3390/molecules31193371)
Supplement: Supplementary file 1 [file molecules-31-03371-s001.zip › molecules-4545584-supplementary.pdf]

Supplementary Materials for

Tip-Sonication-Induced Dilute Alloying of Metal Pairs with Limited Bulk-Immiscibility

Hochan Ahn[1], Hojin Ahn[2,*]

1. Independent Researcher, Hwaseong-si 18472, Gyeonggi-do, South Korea
2. Department of Chemistry, Konkuk University, Seoul 05029, South Korea

* Correspondence: hojinahn@konkuk.ac.kr

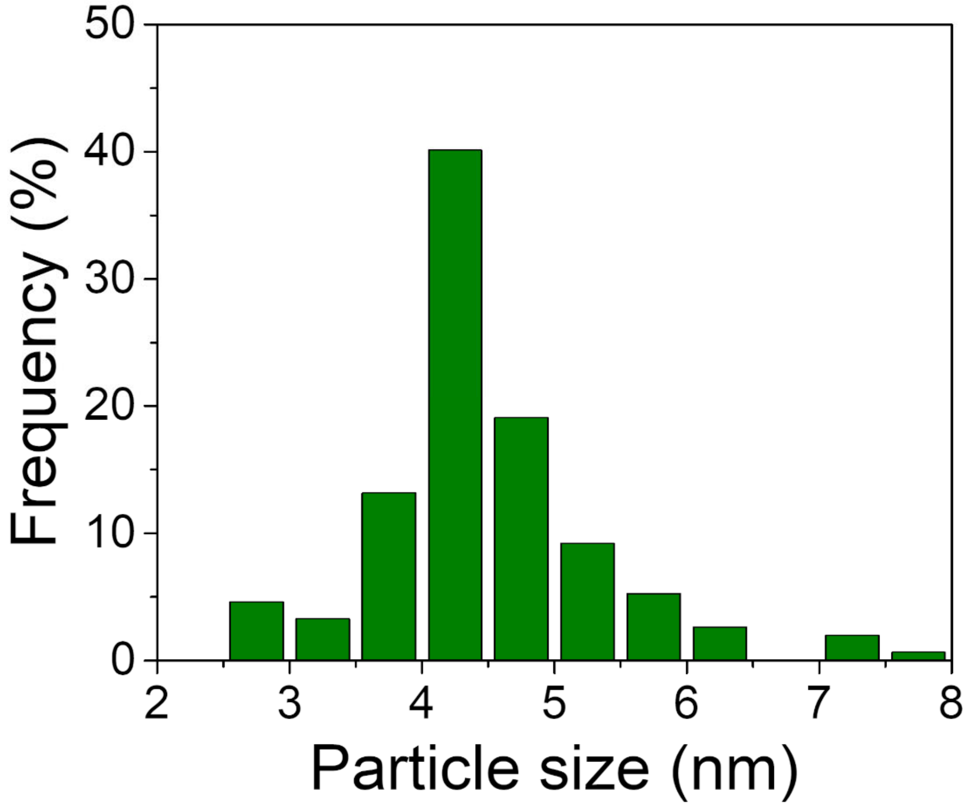

Figure S1. Particle size distribution of the AuMo NPs. The histogram was constructed from measurements of 152 nanoparticles obtained from three independently prepared synthesis batches.

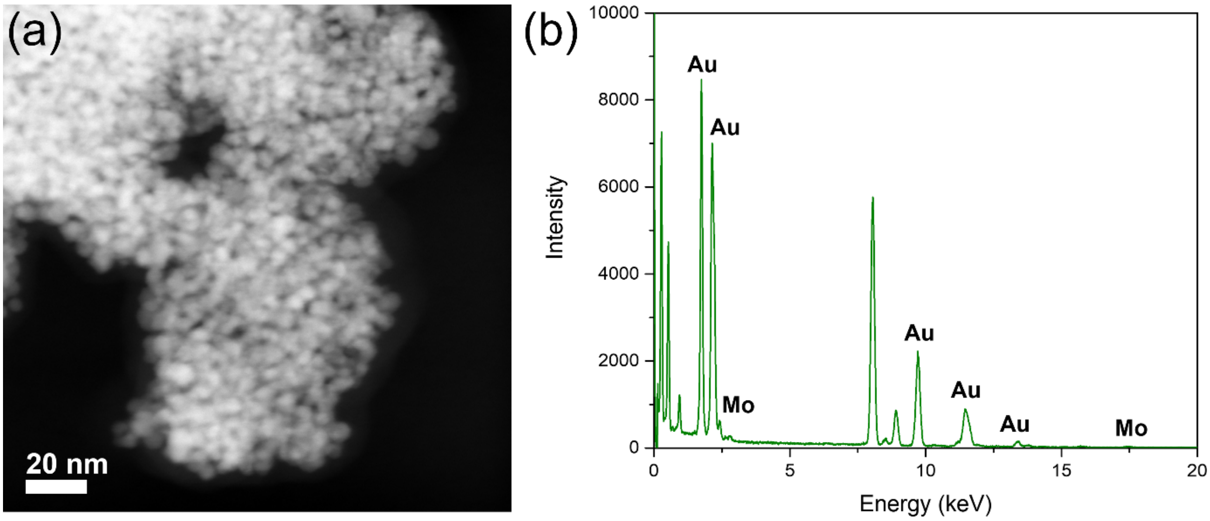

Figure S2. (a) Representative HAADF-STEM image and (b) corresponding EDS spectrum of the AuMo nanoparticles.

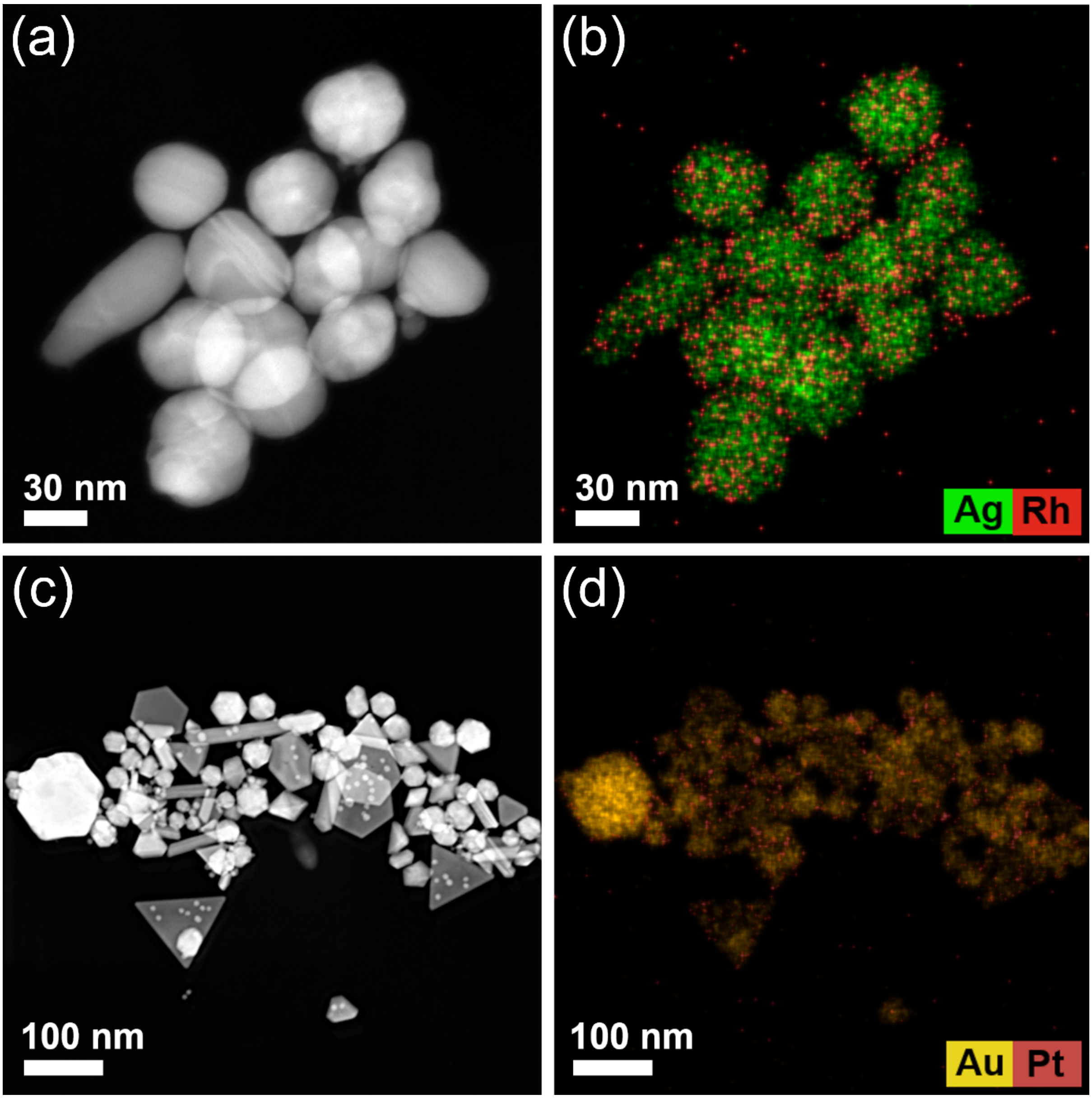

Figure S3. (a) HAADF-STEM image of Ag–Rh nanoparticles acquired at lower magnification and (b) corresponding EDS elemental mapping image. (c) HAADF-STEM image of Au–Pt nanoparticles acquired at lower magnification and (d) corresponding EDS elemental mapping image.
